# Cytoplasmic PCNA is located in the actin belt and involved in osteoclast differentiation

**DOI:** 10.18632/aging.103434

**Published:** 2020-06-27

**Authors:** Donge Tang, Xiaohui Liu, Kezhi Chen, Zhipeng Li, Yong Dai, Jiake Xu, Huan-Tian Zhang, Xuejuan Gao, Langxia Liu

**Affiliations:** 1 Key Laboratory of Functional Protein Research of Guangdong Higher Education Institutes and MOE Key Laboratory of Tumor Molecular Biology, Institute of Life and Health Engineering, Jinan University, Guangzhou 510632, China; 2 Department of Clinical Medical Research Center, The Second Clinical Medical College of Jinan University, The First Affiliated Hospital Southern University of Science and Technology, Shenzhen People’s Hospital, Shenzhen 518020, Guangdong, China; 3 School of Pathology and Laboratory Medicine, University of Western Australia, Perth 6009, Western Australia, Australia; 4 Institute of Orthopedic Diseases and Department of Bone and Joint Surgery, The First Affiliated Hospital, Jinan University, Guangzhou 510630, Guangdong, China

**Keywords:** PCNA, actin belt, cytoplasm, osteoclast, differentiation

## Abstract

Osteoporosis (OP) is an age-related osteolytic disease and characterized by low bone mass and more prone to fracture due to active osteoclasts. Proliferating cell nuclear antigen (PCNA) has been long identified as a nuclear protein playing critical roles in the regulation of DNA replication and repair. Recently, a few studies have demonstrated the cytoplasmic localization of PCNA and its function associated with apoptosis in neutrophil and neuroblastoma cells. However, the involvement of PCNA, including the cytoplasmic PCNA, in the osteoclast differentiation remains unclear. In the present study, we show that PCNA is translocated from nucleus to cytoplasm during the RANKL-induced osteoclast differentiation, and localized in the actin belt of mature osteoclast. Knockdown of PCNA significantly affected the integrity of actin belt, the formation of multinucleated osteoclasts, the expression of osteoclast-specific genes, and the *in vitro* bone resorption. Interactomic study has revealed β-actin as the major interacting partner of the cytoplasmic PCNA, suggesting that cytoplasmic PCNA might play a critical role in the differentiation of osteoclast through regulation of actin-cytoskeleton remodeling. Taken together, our results demonstrate the critical role of cytoplasmic PCNA during the process of osteoclast differentiation, and provided a potential therapeutic target for treatment of osteoclast-related bone diseases.

## INTRODUCTION

With the populations of many countries aging, osteoporosis (OP) has become a major health issue of concern [1]. Just in the years of 2006 and 2007, more than 200 million women in the world were affected by OP, which led to more than 8.9 million fractures every year and seriously worsened the quality of life of the patients [2]. Bone homeostasis is maintained by the balance between bone resorption and formation. OP is caused by excessive bone resorption over bone formation, resulting in reduced bone density and an increased risk of fractures [3]. Opposite to the promotive role of osteoblasts on bone formation, osteoclasts are mainly responsible for bone resorption and bone loss [4]. Thus, revealing the mechanism of osteoclast differentiation would provide ideas for the treatment of osteoclast related or bone destruction specific diseases.

Osteoclasts are giant multinucleated cells with bone-resorbing activity. The osteoclast differentiation is a complex and tightly regulated process characterized by a step of cell fusion amongst the mononuclear precursors to form the mature multinucleated differentiated cells [5]. The osteoclast differentiation can be induced by two key cytokines, namely M-CSF (Macrophage Colony-Stimulating Factor) and RANKL (Receptor activator of nuclear factor kappa-B ligand) [6–8]. Particularly, RANKL has been reported to activate a multitude of signaling pathways and factors required for the differentiation of osteoclasts. IKK (IκB kinase), NF-κB (nuclear factor κB), JNK (c-Jun N-terminal kinase), Akt, c-Src, p38, ERK (Extracellular signal-regulated kinases), AP-1 (activator protein 1), and NFATc1 (nuclear factor and activator of transcription) have all been shown to be the downstream effectors of RANKL during the osteoclast differentiation [9–11]. Among them, NFATc1 is considered to be the master regulator of osteoclast differentiation owing to its activation in the terminal stage of differentiation process and its regulatory function on the expression of osteoclast-specific genes [7, 12–16].

During the osteoclast differentiation, the cytoskeleton plays crucial roles in various cellular functions such as adhesion, migration, signaling transduction and fusion of the differentiating cells. Especially, podosome, the characteristic subcellular structure rich in actin and actin-associated proteins has been shown to be important for the formation and resorbing activity of the osteoclast [17, 18]. During the various stages of the osteoclast differentiation process, podosomes are organized into different patterns: patches, rosettes, actin belt or sealing zone [19, 20]. Actin belt and sealing zone are similar ring-like structures located on the ventral surface of mature osteoclasts. While actin belt are commonly observed in cells adhering both on glass surface and mineralized matrix, sealing zone is reported to be mainly found in osteoclasts attached to the mineralized matrix, with a more compact formation of podosomes allowing the efficient formation of isolated sealing zone of resorption from the enviornment [21–23].

PCNA (proliferating cell nuclear antigen) has been since its discovery identified as a crucial factor of DNA replication and repair, serving as a molecular platform that coordinates the complex interacting network around the DNA polymerases at the replication fork [24, 25]. As such, it is generally considered to be a marker of cell proliferation and a typical nuclear protein. However, during the past few years, it has been revealed that PCNA can act as a cytoplasmic platform controlling the survival of human neutrophil cells through the regulation of their apoptotic process [26–28]. Further, cytoplasmic PCNA connects glycolysis and cell survival in acute myeloid leukemia, echoing the significance of the interaction of cytoplasmic PCNA with components of glycolysis and cancer [27, 29, 30]. Evidence of cytoplasmic PCNA also comes from a study showing the impact of the S-nitrosylation status of PCNA on the apoptotic pathway in a Parkinson disease cell model, and a more recent paper reporting that interaction of cytoplasmic PCNA with angiogenin [31, 32]. These interesting findings hint on the possibilities of the cytoplasmic PCNA exerting other possibly undiscovered functions under other physiological conditions.

In this study, we have observed a striking translocation of PCNA from nucleus to cytoplasm during the RANKL-induced osteoclast differentiation. We strive to determine the precise subcellular distribution of the cytoplasmic PCNA and to investigate its functional importance in the osteoclast differentiation, together with the related molecular mechanism.

## RESULTS

### RANKL induced the translocation of PCNA from nucleus to cytoplasm

**Figure 1:**
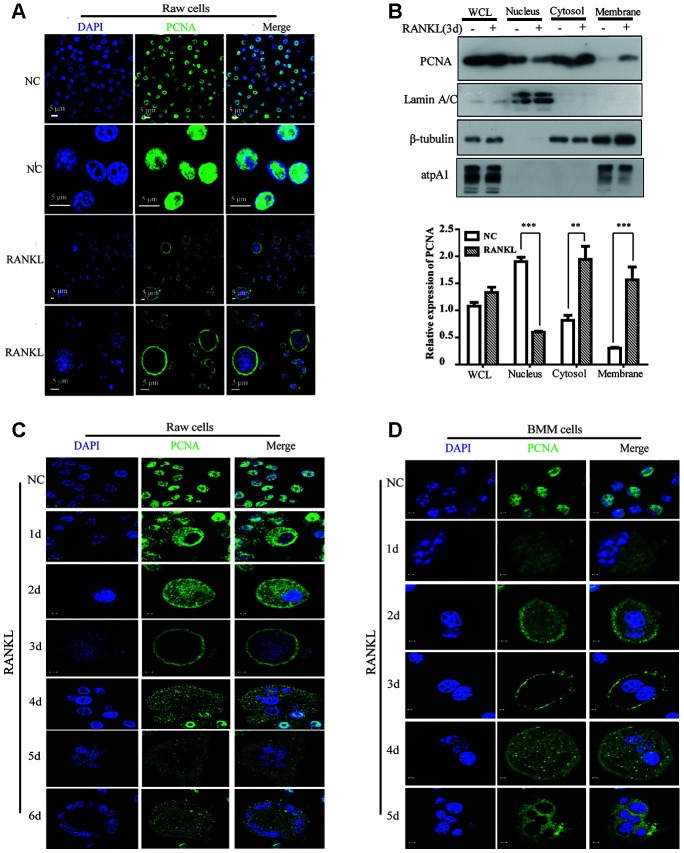
**The subcellular localization of PCNA during the RANKL-induced osteoclast differentiation.** (**A**) Nucleus-cytoplasm translocation of PCNA after 3-day RANKL treatment. RAW264.7 cells were treated with or without RANKL (100 ng/mL) for 3 days to induce osteoclast differentiation. Then, the cells were performed IF assay to test the cellular location of PCNA using primary PCNA antibody. DAPI staining was used to mark cell nucleus. Scale bar: 5μm. (**B**) PCNA subcellular distribution in differentiated osteoclast analyzed by cell fractionation assay. Upper panel: cell fractionation experiments were performed with RAW264.7 previously treated with or without RANKL (100 ng/mL) for 3 days to induce osteoclast differentiation. PCNA protein in various cell fractions was detected by western blotting. Lamin A/C, β-tubulin and atpA1 were respectively used as markers of nucleus, cytosol, and membrane fractions. Lower panel: the quantification of PCNA expression in WCL, Nucleus, Cytosol, and Membrane fractions of three independent experiments. (**C**) Tracking of PCNA subcellular localization during the 6-day RANKL treatment of RAW264.7 cells. IF assays were performed with RAW264.7 cells treated with or without RANKL (100 ng/mL) for the indicated times of treatment. Scar bar: 2-5μm. (**D**) Tracking of PCNA subcellular localization during the 5-day RANKL treatment of BMM cells. After isolation, BMM cells were cultivated with M-CSF (10 ng/mL) and RANKL (100 ng/mL) for 5 days to induce the osteoclast differentiation, and analyzed as described in (C). Scar bar: 2-5μm.

Previous studies have demonstrated that cytoplasmic PCNA plays important roles in the regulation of apoptosis in differentiated neutrophils [26–28]. We tried to examine the subcellular localization of PCNA in differentiated osteoclast and investigate its function. To this end, we firstly performed confocal immunofluorescence assay in undifferentiated RAW264.7 cells and in mature RAW264.7 cells induced by RANKL. Figure 1A showed that PCNA was mainly localized in the nucleus of undifferentiated RAW264.7 cells. However, in those cells treated with 100 ng/mL RANKL during three days, a large number of PCNA has been translocated from nucleus to cytoplasm, precisely in the cell periphery, forming a ring-like structure at the proximity of plasma membrane (Figure 1A). In order to further confirm the nucleus-cytoplasm translocation of PCNA during osteoclast differentiation, cell fractionation experiment was performed to separate the fractions of cell nucleus, cytoplasm and cell membrane. Western blot assay was then used to analyze the expressional levels of PCNA in these three components before and after RANKL induction. The results of Figure 1B showed that the total expression of PCNA was not significantly changed before and after RANKL induction. Compared with the control group, the nuclear PCNA was significantly reduced in RAW264.7 cells after 3-day RANKL treatment, while PCNA in the cytoplasmic and membrane fractions was significantly increased, indicating that PCNA was indeed translocated from the nucleus to the cytoplasm and, moreover, associated with cell membrane during the process of RANKL-induced osteoclast differentiation.

In order to observe the dynamic process of PCNA translocation during osteoclast differentiation, RAW264.7 cells were stimulated with RANKL during five or six days, and the subcellular localization of PCNA was recorded daily by confocal immunofluorescence. As shown in Figure 1C, during the first three days of RANKL induction, we have been able to observe a gradual translocation of PCNA protein from the nucleus to the cytoplasm and then to the plasma membrane, with the most marked plasma membrane localization on the third day. Interestingly, at day 4, day 5 and day 6, PCNA on the plasma membrane was gradually relocated to the cytoplasm, in a punctual staining pattern (Figure 1C). These results were then reproduced in the mouse primary macrophage BMM cells in which we have been able to observe a similar dynamic process of translocation of PCNA protein induced by RANKL treatment (Figure 1D).

### PCNA knockdown affected the RANKL-induced differentiation of osteoclast

**Figure 2:**
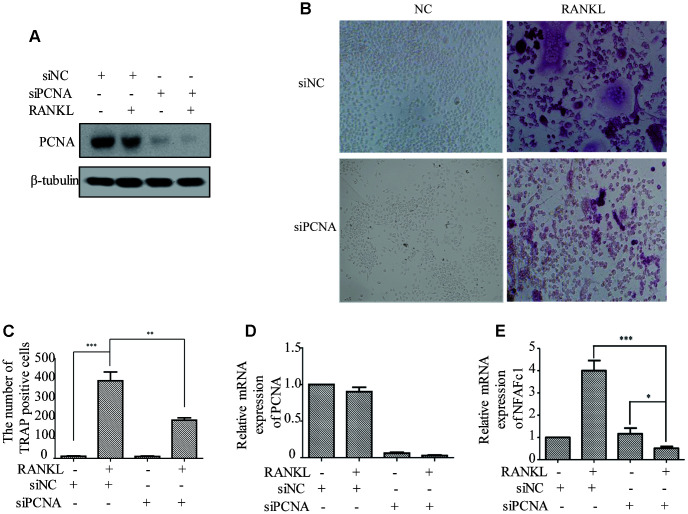
**The effect of PCNA knockdown on RANKL-induced differentiation of osteoclast.** (**A**) PCNA was successfully knocked down using its siRNA. RAW264.7 cells were transfected with siRNA targeting PCNA for 48 hours. Then, the cells were treated with RANKL (100 ng/mL) for 3 days and harvested for western blot assay using the indicated antibodies. (**B**) The effect of knockdown of PCNA on osteoclast differentiation. RAW264.7 cells were transfected with PCNA siRNA for 48 hours, then the cells were treated with RANKL for 3 days and stained with TRAP. (**C**) The number of TRAP-positive cells stained in (**B**). n=5, **: p <0.01, ***: p<0.001. Then, the expression of osteoclast differentiation marker NFAFc1 was detected in control and RANKL-induced RAW264.7 cells, with or without PCNA knockdown (**D**, **E**). *: p<0.05, ***: p<0.001.

In order to investigate the functional significance of PCNA translocation of PCNA during osteoclast differentiation, we used PCNA-targeting siRNA to knock down its expression in RAW264.7 cells, and examine the effect on the RANKL-induced differentiation of these cells. RAW264.7 cells transfected with PCNA siRNA or control siRNA and treated with RANKL for three days were analyzed using TRAP staining to determine the stage of differentiation. The successful knockdown of PCNA expression was firstly confirmed by western blotting (Figure 2A). Results of the TRAP staining assays demonstrated that, after 3-day RANKL induction, the differentiation of PCNA-knockdown cells was significantly blocked as compared with those transfected with the control siRNA (Figure 2B and 2C). The mRNA level of NFATc1 which is considered as the master regulator and a marker of osteoclast differentiation was further examined in these cells by qRT-PCR to confirm the effect of PCNA knockdown on osteoclast differentiation [15, 16]. The results in Figure 2D and 2E showed that the knockdown of PCNA significantly reduced the expression level of NFATc1 induced by RANKL treatment. Taken together, these results suggested that PCNA is required for osteoclast differentiation induced by RANKL.

**Figure 3:**
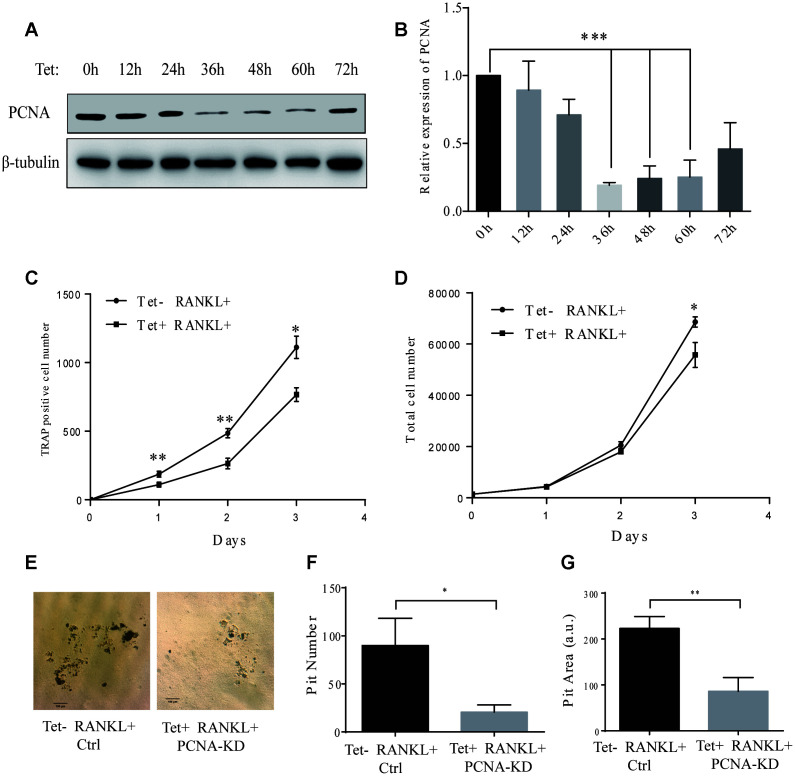
**Effect of inducible knockdown of PCNA on RANKL-induced osteoclast differentiation.** (**A**) The time course of PCNA knockdown effect after Tet-induction. 20 μg/mL of Tet was added Tet-PCNA KD cells to induce the knockdown of PCNA. Cells were harvested at different time points as indicated (0-72 hours) and subjected to western blotting to assess the effect of PCNA knockdown. (**B**) The quantification of PCNA expression level presented in (**A**). ***: p<0.001. (**C**) The impact of PCNA knockdown after its nuclear-cytoplasmic translocation on RANKL-induced osteoclast differentiation. Tet-PCNA KD cells were simultaneously treated with both Tet and RANKL for 3 days. TRAP-positive cells (considered as differentiated cells) were counted each day. (**D**) The effect of knockdown of cytoplasmic PCNA on the proliferation of RAW264.7 cells. The total cell number of cells after same treatments as in (**C**) was counted. *: p<0.05, **: p<0.01. (**E**) *In vitro* bone-resorption assays. Tet-PCNA KD cells were seeded at Corning Osteo Assay Surface multiple well plates and treated with Tet (20 μg/mL), RANKL (100 ng/mL) and M-CSF (50 ng/mL) for 10 days to induce the knockdown of PCNA, differentiation of osteoclasts and osteoclast-mediated bone resorption. Tet-PCNA KD cells treated without Tet were set as control. Resorption pits were observed using an Olympus microscope at 25x magnification. Scale bar: 100 μm. (**F**) Quantification of the resorption pits. Resorption pits on each well were counted. The results presented are the means ± SD of three independent experiments. *: p<0.05. (**G**) Quantification of the area of total pits. The area of total pits on each well was analyzed by ImageJ software. The results presented are the means ± SD of three independent experiments. **: p<0.01.

In the PCNA knockdown experiments described hereinabove, since PCNA was knocked down before RANKL induction, and considering the critical role of PCNA in cell proliferation, it remained unclear whether the differentiation failure observed was the consequence of the proliferative alteration due to the loss of nuclear PCNA, or the manifestation of the loss of function of cytoplasmic PCNA, which would directly affect the differentiation process. In order to address this issue, we have established a RAW264.7 cell line with a Tet-on inducible lentiviral expression vector to knock down PCNA in a time-controlled manner. The efficiency and time course of PCNA knockdown in such cells (hereinafter referred as to Tet-PCNA KD cells) was firstly checked. Figure 3A and 3B showed the time course of PCNA knockdown effect after Tet-induction. As shown, 36 hours after Tet-induction, PCNA expression was significantly suppressed, and the effect lasted for the rest of the examination period (36-72 hours after Tet-induction). Based on this observation, we then combined Tet and RANKL treatments to examine the effect of PCNA knockdown mainly occurred after its nuclear-cytoplasmic translocation during the RANKL-induced osteoclast differentiation. Since PCNA translocation could be observed as early as 24 hours after RANKL induction in our precedent experiments, and PCNA knockdown took effect 36 hours after Tet-induction, we added simultaneously Tet and RANKL to the Tet-PCNA KD cells to as far as possibly ensure the further knockdown of PCNA mainly after its translocation to cytoplasm. Total cell number and TRAP-positive cell number were recorded every 24 hours after treatments in order to evaluate the proliferation potential and differentiation stage of cells. Tet-PCNA KD cells treated only with RANKL but not with Tet were used as a control. Our results in Figure 3C-D showed that little variation of total cell number was observed between Tet-treated and non-Tet-treated group after RANKL induction, especially in the first 48 hours. In contrast, TRAP-positive cell number significantly decreased in Tet-treated group as compared with control group, suggesting that the knockdown of PCNA protein after its nuclear-to-cytoplasmic translocation in the differentiating cells significantly inhibited the differentiation process, but had little influence on cell proliferation. Moreover, knockdown of PCNA protein after its nuclear-to-cytoplasmic relocalization significantly reduced the ability of *in vitro* bone resorption mediated by osteoclasts (Figure 3E-G). This result clearly supported our hypothesis that, in addition to the more classic role in cell proliferation of nuclear PCNA, the cytoplasmic PCNA may be involved in the differentiation of osteoclast.

### Identification and analysis of the proteins interacting with cytoplasmic PCNA

**Figure 4:**
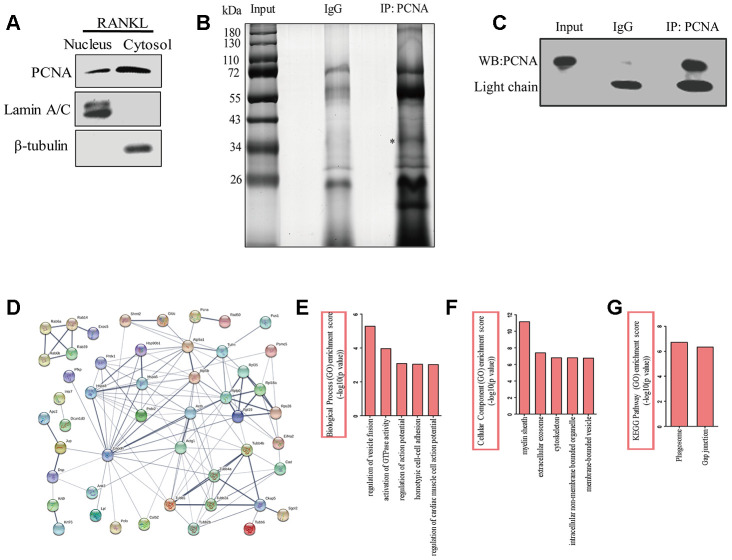
**The identification of the proteins interacting with cytoplasmic PCNA.** (**A**) RAW264.7 cells after RANKL (100 ng/mL) induction for three days were subjected to fractionation experiments to isolate the nuclear and cytoplasmic fractions. The distribution of PCNA in both fractions was assessed by western blotting. Lamin A/C and β-tubulin were respectively used as markers for nuclear and cytoplasmic fractions. (**B**) Co-IP assay using PCNA antibody was carried out with the cytoplasmic fraction, PCNA-bound proteins were separated using SDS-PAGE gel electrophoresis and silver stained. IP with IgG was used as the control. The asterisk denotes the protein band corresponding probably to the immunoprecipitated PCNA protein. (**C**) Confirmation by western blotting of the successful immunoprecipitation of PCNA in (**B**). Light chain: the light chain of IgG and PCNA antibody. (**D**) STRING program analysis of the interaction network of 76 putative cytoplasmic PCNA interactors. Line thickness indicates the strength of data support. (**E**) Biological processes analysis of the identified differential proteins using the ClueGO plug-in of Cytoscape software. The top five items were listed. (**F**) Cellular component enrichment analysis of the identified differential proteins using Cytoscape software. The top five items were listed. (**G**) KEGG pathway enrichment analysis of the identified differential proteins using Cytoscape software. The top two items were listed.

In order to better understand the function of cytoplasmic PCNA in osteoclast differentiation, and elucidate the related molecular mechanism, we tried to identify the interacting partners of cytoplasmic PCNA. To this end, the nuclear and cytoplasmic fractions of RANKL-induced RAW264.7 cells were firstly separated by cell fractionation. Western blotting shown in Figure 4A confirmed the distribution of PCNA in the nuclear and cytoplasmic fractions, in which Lamin A/C and β-tubulin were respectively used as the nuclear and cytoplasmic markers. Immunoprecipitation experiment was then carried out with the cytoplasmic fraction using an anti-PCNA antibody. Proteins co-immunoprecipitated with PCNA were separated by SDS-PAGE and dyed with silver staining (Figure 4B). The differential bands compared with the control IgG group, including specific protein band (denoted by the asterisk in Figure 4B), in the PCNA-IP lane were subsequently, analyzed by LC-MS/MS for their identification. The results of our analyses showed that 14 unique peptides of PCNA protein (data not shown) were identified in the band marked with asterisk, demonstrating the successful immunoprecipitation of PCNA, that has also been confirmed by western blotting assay with an anti-PCNA antibody (Figure 4C).

Proteins identified in the PCNA-IP lane were subsequently compared with that identified in the control lane. Those found in both lanes were considered to be non-specific binders and excluded from further studies. This resulted in the identification of a total of 76 proteins considered as the putative interactors of cytoplasmic PCNA for further analyses (Supplementary Table 1). First, online STRING program (https://string-db.org/) was used to build a protein interaction network comprising these putative interactors and PCNA (Figure 4D). With the total 77 (including 76 putative proteins and PCNA) protein names inputted, 3 were not recognized by the program and excluded from the analysis. Among the 74 remaining proteins analyzed (nodes), the number of interactions (edges) was 133, significantly superior to the expected value that is 60, resulting in an average node degree of 3.64, a clustering coefficient of 0.45, and a PPI enrichment p-value of 5.55e^-16^. These data indicate that the protein interaction network is statistically reliable and functionally relevant. These proteins were then grouped and annotated based on their GO (Gene Ontology) using the ClueGO plug-in of Cytoscape software. The most enriched biological processes include the regulation of vesicle fusion, the activation of GTPase activity, the regulation of action potential, the homotypic cell-cell adhesion, and the regulation of cardiac muscle cell action potential, ranked according to their enrichment scores (-log10 (p-value), Figure 4E). When annotated based on their subcellular localization, these proteins were found to be associated with myelin sheath, extracellular exosome, cytoskeleton, intracellular non-membrane bounded organelle, and membrane-bounded vesicle (Figure 4F). Furthermore, KEGG analysis showed that phagosome and gap junction were the leading pathways involved (Figure 4G). Taken together, the results of these analyses strongly suggested the association of these proteins with the cellular functions such as vesicle fusion, phagocytosis, and cytoskeleton dynamics.

### Cytoplasmic PCNA interacts with ACTB and is necessary for the integrity of actin belt

**Figure 5:**
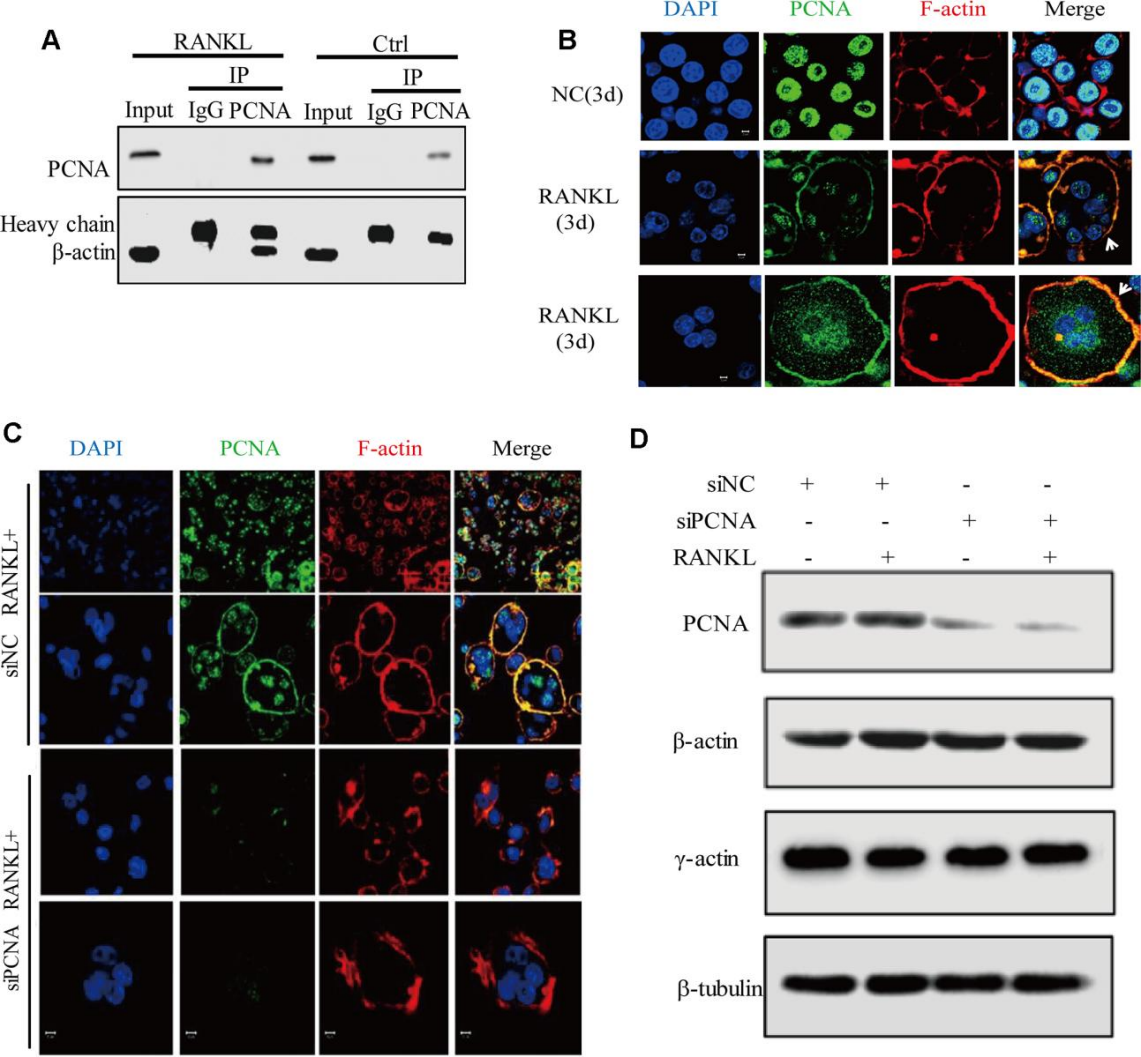
**Cytoplasmic PCNA interacts with β-actin and is necessary for the integrity of actin belt.** (**A**) Interaction between PCNA and β-actin in RANKL-induced RAW264.7 cells. RAW264.7 cells with or without RANKL (100 ng/mL) induction for three days were used to perform co-IP assay using IgG and primary PCNA antibody. Western blot assay was then carried out to detect PCNA and β-actin in the immune complexes. Heavy chain: the heavy chains of control IgG and PCNA antibody. (**B**) The subcellular localization of PCNA and F-actin. RAW264.7 cells with or without RANKL (100 ng/mL) induction for three days were used to perform IF experiments. PCNA was labeled by Alexa 488 (green). F-actin was dyed with phalloidine-conjugated Alexa Fluor 594 (red). The images of the two bottom panels were two different representative osteoclasts. Scar bar: 5μm. (**C**) The effect of knockdown PCNA on the integrity of actin belt. RAW264.7 cells were transfected with siRNA targeting PCNA for 48 hours, then, the cells were treated with RANKL (100 ng/mL) for 3 days and IF assay were performed to assess the integrity of actin belt. Scar bar: 5μm. (**D**) The effect of PCNA knockdown on the expressions of β-actin and γ-actin. RAW264.7 cells were transfected with PCNA siRNA and treated with RANKL (100 ng/mL) for 3 days. Then, the cells were harvested and lysed for standard western blot assay using the indicated antibodies.

Among the putative interactors of the cytoplasmic PCNA identified as described hereinabove, the best scored one is ACTB (β-actin), one of the building blocks of actin-cytoskeleton (Supplementary Table 1). Based on the critical role of actin-cytoskeleton in the process of osteoclast differentiation, especially in the formation and dynamics of podosomes, we hypothesized that cytoplasmic PCNA might be involved in osteoclast differentiation through interaction with β-actin. To confirm the interaction of PCNA with β-actin during the process of osteoclast differentiation, the standard co-immunoprecipitation assay was carried out in RAW264.7 cells with or without 3-day RANKL treatment. The result in Figure 5A showed that β-actin co-immunoprecipitated with PCNA in RANKL-treated cells but not in untreated control cells. Moreover, we also confirmed the association of β-actin with cytosolic PCNA using co-IP assay in the cytosolic fraction of RANKL-induced RAW264.7 cells (Supplementary Figure 1A, 1B) and the direct interaction between GST-β-actin and His-PCNA using GST-pulldown (Supplementary Figure 1C). In view of our confocal immunofluorescence results showing that PCNA in untreated cells is exclusively nuclear while mostly cytoplasmic in RANKL-induced cells (Figure 1), this suggested that β-actin is an interacting partner for the cytoplasmic PCNA but not for the nuclear PCNA. The physical interaction between cytoplasmic PCNA and β-actin, together with our observation that PCNA is located at the proximity of plasma membrane in a ring-like structure in osteoclast (Figure 1), prompted us to speculate the localization of cytoplasmic PCNA in the actin belt. Confocal immunofluorescence assays were performed to verify this hypothesis. As expected, in RAW264.7 cells induced with RANKL for 3 days, we have been able to observe the perfect overlap of PCNA staining with F-actin dyed with phalloidin, indicating that the cytoplasmic PCNA is indeed a protein located in the actin belt (Figure 5B).

To understand the functional significance of PCNA and β-actin interaction, we assessed the effect of PCNA knockdown on the superstructure of F-actin during osteoclast differentiation in the RAW264.7 cells transfected with siRNA of PCNA (Figure 5C). Immunofluorescence analysis of F-actin indicated that, while most control cells possessed intact closed ring-like actin belt, PCNA-knocked down cells displayed truncated actin belt with irregular patch-like staining pattern, suggesting that PCNA knockdown affected the integrity of actin belt in RANKL-induced osteoclasts (Figure 5C). Moreover, western blotting shown in Figure 5D revealed that PCNA deficiency had no effect on β- or γ-actin expression. These results suggested that the cytoplasmic PCNA might be involved in the construction and/or maintenance of actin belt that is important for the formation of multinucleated osteoclast through physical interaction with β-actin.

### Co-localization of PCNA with Rab7 in the endosomes and ruffled border-like structure

**Figure 6:**
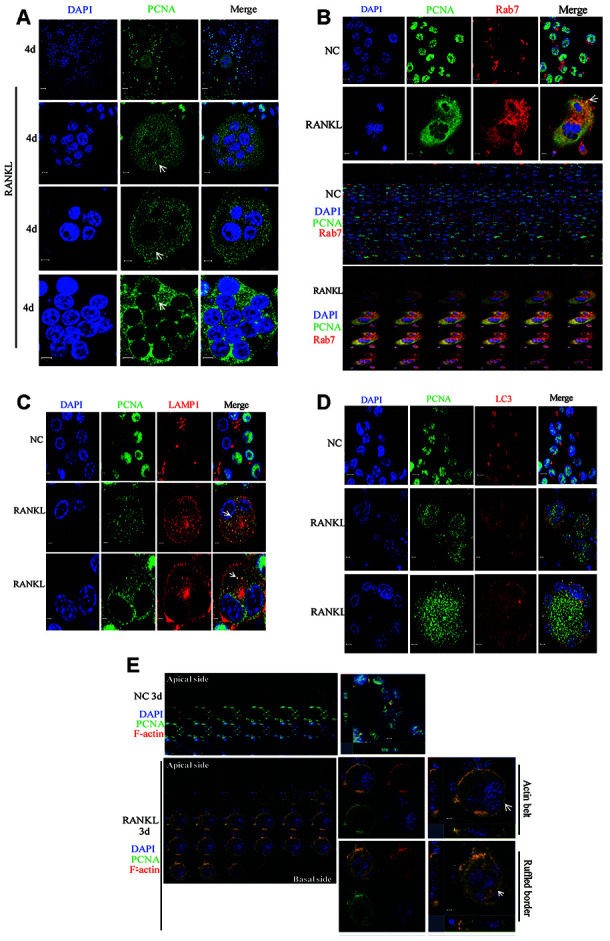
**The co-localization of PCNA with Rab7 in RANKL-induced RAW264.7 cells.** (**A**) IF assay was performed to localize PCNA in the 4-day RANKL-treated RAW264.7 cells using appropriate PCNA antibody. Arrows: PCNA located in the cytoplasm. Scar bar: 20μm for the top panel images and 5μm for the bottom three panels. (**B**) Co-localization of PCNA with Rab7 in RANKL-induced osteoclast. RAW264.7 cells were treated with RANKL (100 ng/mL) for 3 days and subjected to IF assays using PCNA and Rab7 antibodies. The Z-stack scan model of the LSM microscope was used to observe the cells from the abdominal end to dorsal end. All images were presented in order. Arrows: co-localization of PCNA with Rab7. Scar bar: 5μm for negative control (NC) cells and 10μm for RANKL treated cells. (**C**) Co-staining of PCNA with LAMP1 in RANKL-induced osteoclast as described in (**B**). Scar bar: 2μm. (**D**) Co-staining of PCNA with LC3 in RANKL-induced osteoclast as described in (B). Scar bar: 5μm. (E) Z-stack scan mode in confocal microscopy was used to detect the subcellular localization of PCNA and F-actin in the 3-day RANKL treated RAW264.7 cells. Upper panels: untreated negative control RAW264.7 cells; lower panels: 3-day RANKL treated RAW264.7 cells. Scar bar: 5μm.

Conventionally, RAW264.7 or primary BMM cells in culture are treated with RANKL for 3 days to obtain the mature and functionally active osteoclasts. At this time point in our experiments, PCNA is localized mainly in the actin-belt (Figure 1). However, as also shown in Figure 1, at the other time points of RANKL induction, for example, at day 2, day 4 or day 5, cytoplasmic PCNA displayed another pattern of staining in the form of scattered dots (Figure 1C and 1D). In view of this typical vesicle-like staining pattern, we tried to determine whether cytoplasmic PCNA could be located in one of the 3 most common vesicles in the cell, i.e. endosome, lysosome and autophagosome. We have respectively used Rab7, LAMP1 and LC3 as the markers of these three types of vesicles in confocal immunofluorescence assays to examine whether PCNA could be co-localized with any of these three markers. As shown in Figure 6A-6D, in RANKL-treated RAW264.7 cells, cytoplasmic PCNA was co-localized in a decrease order with Rab7, LAMP1 and LC3. While a considerable part of the Rab7 positive vesicles were also stained by PCNA antibody, only a few of those labeled by LAMP1 antibody were PCNA positive, and as contrast, LC3 positive autophagosomes were basically free of PCNA staining. Consistently, our co-IP assay has identified interacting partners of cytoplasmic PCNA including Rab6, Rab14 and Rab39 (Supplementary Table 1). This suggested that, through various steps of osteoclast differentiation induced by RANKL, a part of Rab7-positive late endosomes contain PCNA, which could possibly be the transported cargo between nucleus and plasma membrane, or the functional component participating in the intracellular transport and secretion of other molecules required for the resorbing function of the cell.

Furthermore, in the RAW264.7 cells at day 3 of RANKL induction, during the Z stack analysis by using confocal immunofluorescence method, we have discovered that, in addition to the actin-belt localization mentioned above, cytoplasmic PCNA was also found in a basal/ventral area resembling the ruffled border when viewed along the Z-axis and could also be stained with phalloidin and anti-Rab7 antibody in the literatures [33, 34] (Figure 6E). It has been well documented in the literature that Rab7 is a protein located in the ruffled border and important for the formation of ruffled border and the resorbing function of the osteoclast [33, 35, 36].

## DISCUSSION

**Figure 7:**
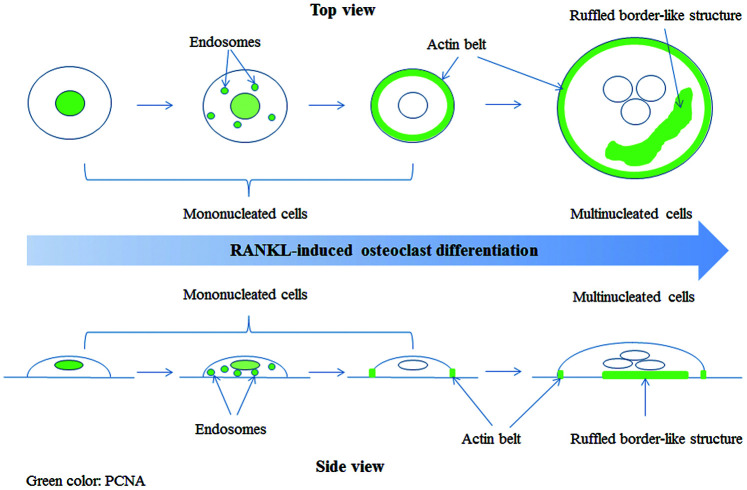
**A model of PCNA subcellular localization (indicated by green color) during RANKL-induced osteoclast differentiation.**

OP is an age-related disease resulted from imbalance in bone homeostasis. Overproduced osteoclasts partly contributed to this imbalance. However, the mechanisms of osteoclast differentiation remain unclear. In this study, we showed that, in addition to its well-known role in cell proliferation, PCNA was involved in the positive regulation of RANKL-induced osteoclast differentiation. Moreover, this novel function of PCNA seems to be associated with its newly discovered cytoplasmic localization different from its classic nuclear distribution necessary for its functions in DNA repair and replication. Interestingly, we observed that during the process of osteoclast differentiation, PCNA was translocated from nucleus to cytoplasm, and localized at the actin belt or ruffled border-like structure of mature osteoclast (Figure 7). Knockdown of PCNA after its nuclear-to-cytoplasmic translocation using an inducible lentiviral system reduced the formation of multi-nucleated osteoclasts and the ability of *in vitro* bone resorption mediated by osteoclasts (Figure 3). Moreover, by interactomics approach, we have identified β-actin as the major interacting partner of the cytoplasmic PCNA in RANKL-induced osteoclasts, further providing the mechanistic basis for the involvement of cytoplasmic PCNA in the formation or/and function of actin belt during the differentiation of osteoclast. Our study not only described a novel physiological function of PCNA as a regulator of cellular differentiation, but also unveiled a new mode of action of PCNA characterized by its spectacular translocation, and its physical interaction with β-actin, one of the building block of cytoskeleton, corroborated by its functional impact on the formation or maintenance of actin-rich structure. Considering the prominent role of actin cytoskeleton in the physiology of cell, this finding is very suggestive regarding the yet uncovered roles of PCNA in other cellular functions or physiological processes beyond the scope of osteoclast differentiation. Up to now, the nuclear-cytoplasmic translocation of PCNA was mainly observed in well-differentiated cells from Dr. Witko-Sarsat ’s work [26&^28^], and our present study, but one could easily imagine that under various physiological and pathophysiological conditions of diverse cells, whenever the translocation of PCNA is triggered, this protein might move to new subcellular compartments, interact with new partners, and exert alternative functions. For example, in the processes of embryonic development, tissue regeneration, senescence or cancer development, where the determination of cell fate is the principal subject, PCNA might be a critical factor not only by its activity in the nucleus but also as a cytoplasmic protein, and by interaction with actin-cytoskeleton. However, PCNA being a predominantly nuclear protein, the conditions required for its nuclear exportation might vary in different cells and processes and are difficult to be determined.

In relation to the molecular mechanism of nuclear-to-cytoplasmic relocalization of PCNA, reported studies indicated that the chromosome region maintenance 1 (CRM1) exportin-dependent nuclear export signal (NES) was involved in PCNA shuttling during granulocytic differentiation [37]. Inhibition of CRM1 with its inhibitor leptomycin B led to the inhibition of PCNA relocalization [37]. Moreover, NES located from Ile-11 to Ile-23 in the PCNA sequence was identified to contribute to this translocation [37]. However, it needs further investigation to test whether PCNA nuclear-to-cytoplasmic relocalization in osteoclasts used a similar mechanism. As to the binding sites on PCNA for its partners, the reported interaction sites included the interdomain-connecting loop (IDCL), spanning residues from L121 to E132, the N-terminal region, and the C-terminal tail of PCNA [24, 38]. Several proteins, such as pol δ, p21 and flap endonuclease 1 (Fen1), interacted with the IDCL domain of PCNA through their canonical PCNA-interacting protein (PIP)-box QXXhXXaa (h is frequently I/L/M, a is F/W/Y, and X is any amino acid) [39]. By checking the amino acid sequence of β-actin, we found that there is a QAVLSLYA sequence, similar to PIP-box with the exception of the last amino acid A, which could possibly be a PIP-box like motif in β-actin responsible for the interaction with the IDCL domain of PCNA. This speculation needs further investigation.

Osteoclast differentiation and function rely heavily on actin cytoskeleton remodeling. The podosome, an actin-rich protrusive structure characteristic to osteoclast, plays critical roles in multiple stages of osteoclast differentiation and also in the resorbing activity of mature osteoclast [17, 18]. In particular, the integrity of actin belt is critical for the formation of mature osteoclast and its resorptive activity, but the underlying mechanisms of its regulation remain incompletely understood nowadays. Several podosome-associated proteins have been previously shown to be important for the integrity of the actin belt. It has been reported that tyrosine kinase Src deficiency caused the disruption of actin belt formation and osteoclast differentiation [40]. Through binding to the cytolinker protein plectin, Src was shown to be co-localized with plectin close to the actin belt and regulated actin belt formation and osteoclast differentiation [21]. Knockdown of another cytolinker protein, microtubule actin crosslinking factor 1 (MACF1), also disrupted actin belt formation and further blocked the bone resorption activity of osteoclasts through Akt/GSK3β/NFATc1 signalling pathway [41]. In the present study, we observed that PCNA knockdown affected dramatically the integrity of actin belt. PCNA knockdown led to the truncated actin belt structure with patch-like staining of F-actin (Figure 5). This observation suggested that PCNA might be involved in the formation or/and maintenance of actin belt. In order to clarify the mechanism of action of such function, we carried out the interactomic study of cytoplasmic PCNA, and revealed β-actin as the principle interactor of cytoplasmic PCNA (Supplementary Table 1, Figure 5). Moreover, GO analysis of the identified 76 potential partners suggested the most enriched biological processes include the regulation of vesicle fusion, the activation of GTPase activity, and the regulation of action potential (Figure 4E). Finally, immunofluorescence experiments indicated that cytoplasmic PCNA was perfectly co-localized with F-actin in the actin belt (Figure 5B). All these data suggest that PCNA might serve as an adaptor or scaffold protein connecting actin with other podosome-associated proteins.

It has been reported that bone resorption activities of osteoclasts included the activation of a variety of intracellular membrane trafficking pathways. Small GTP-binding proteins of Rab family are well known key regulators of membrane trafficking pathways [42]. For example, Rab7 and Rab9 localized at the late endosome and regulated the corresponding trafficking events [35, 43]. In osteoclasts, Rab7 and Rab9 have been reported to localize at the ruffled border membrane (a late endosomal-like compartment in the plasma membrane) in non-resorbing osteoclasts cultured on glass coverslips, which suggested Rab7 might regulate the targeting and fusion of late endosomes with the ruffled border membrane and late endocytotic pathways that might be involved in the secretion of lysosomal enzymes, such as cathepsin K [44]. Recently, Rab7 has been reported to be associated with the trafficking of secretory lysosomes in osteoclasts for bone resorption [45]. In our study, PCNA and Rab7 was co-localized both in vesicle-like and ruffled border like structures (Figure 6), hinting on the possibility that the cytoplasmic PCNA, as like Rab7, might also play a role in the trafficking between late endosomes and ruffled border membrane (or plasma membrane) of osteoclasts for the exocytosis and/or endocytosis functions necessary for resorptive activities of the cells. Interestingly, we have identified other Rab family membranes including Rab6, Rab14 and Rab39 interacting with cytoplasmic PCNA (Supplementary Table 1), suggesting that cytoplasmic PCNA might participate in various vesicle trafficking. Indeed, the expressions of several Rab proteins containing Rab6, Rab7 and Rab14 have been identified in osteoclasts [46, 47]. And the prenylation of Rab2B, Rab3D, Rab5, Rab6, Rab7 and Rab14 have been reported to be involved in the osteoclast resorptive function [47]. Further investigation aiming to clarify the roles of these Rab proteins in relation with the function of cytoplasmic PCNA would be interesting.

Taken together, our study described for the first time the dynamic translocation of PCNA from nucleus to cytoplasm during the differentiation of osteoclasts and its localization in the actin belt, a critical structure for the osteoclast differentiation and function. Using the inducible PCNA knockdown cell model, we further demonstrated the important role of the cytoplasmic PCNA in osteoclast differentiation. These results would provide a potential therapeutic target of cytoplasmic PCNA for the therapy of osteoclast-related bone diseases like OP.

## MATERIALS AND METHODS

### Cell culture

RAW264.7 macrophages (ATCC, USA) were cultured in alpha modified of Eagles Medium (α-MEM, Gibco BRL, Grand Island, NY) supplemented with 10% fetal bovine serum (FBS, PAN-Biotech Germany). Cells were maintained at 37 °C in an incubator (HERAcell240i, Thermo, USA) with a humidified atmosphere containing 5% CO_2_. The cells are free from mycoplasma infections tested with the commercialized TransDetect® Luciferase Mycoplasma Detection Kit (TransGen Biotech Co., LTD, Beijing, China). To induce the differentiation of RAW264.7 cells into osteoclasts, 100 ng/ml of RANKL was added to the cells for three more days with changing media every two days as described previously [12].

### Isolation and culture of primary bone marrow-derived macrophages (BMM)

The male mice of 6 weeks old were executed by cervical dislocation. Then, the femur and tibia were extracted as described previously [12]. Briefly, after disinfection and cutting of both ends of these bones, DMEM was repeatedly used to flush the bone marrow as thoroughly as possible. The cell debris was filtered with 70 μm nylon mesh. Then, centrifugation with 250 g was carried out to collect the precipitate. 2 mL ACK (Ammonium-Chloride-Potassium) Lysing Buffer was added to the precipitate for 2 min to lyse erythrocytes. After centrifugation with 250 g again, the BMM cells in the precipitate were collected and cultured with DMEM containing 10 % fetal bovine serum for subsequent experiments. M-CSF (10 ng/mL) and RANKL (100 ng/mL) treatment for three or more days were used to induce the osteoclast differentiation of BMM cells. All experiments were performed according to the approved guidelines and all animal experimental protocols were approved by the animal experimental ethics committee of Jinan University.

### Small interfering RNA assays

The sequences of siRNA used in this study are as follows: PCNA siRNA: 5’-GCCUGUUCACCUAACGUUUTT-3’ and 5’-AAACGUUAGGUGAACAGGCTT-3’, and control siRNA (NC): 5’-UUCUCCGAACGUGUCACGUTT-3’ and 5’-ACGUGACACGUUCGGAGAATT-3’. Transfection of siRNA was performed using Lipofectamine™ 2000 (Invitrogen, Carlsbad, CA) according to the manufacturer’s instructions.

### Screening RAW264.7 cell lines with a Tet-on inducible PCNA knockdown

Firstly, we constructed a Tet-on inducible lentiviral expression vector to knock down PCNA. For this purpose, the pTRIPZ (Obio Technology (Shanghai) Corp.,Ltd., China) was used. The small interference sequence for PCNA was inserted into pTRIPZ to produce pTRIPZ-PCNA.

293T cells at a 80-90% confluence were co-transfected with pLP1, pLP2, pLP-VSVG, pTRIPZ-PCNA plasmids (the ratio is 1:1:1:2) using lipofectamine 2000. After expression for 48-72 hours, the culture medium of the cells was collected through a centrifuge for 10 min at 3500 rpm. The produced lentivirus was collected after filtration with 0.45 μm filter membrane. Then 2 mL of this culture medium together with 10 μL polybrene (1 mg/mL) were added into RAW264.7 cells.

After 72 hours, 10 μL puromycin (1 μg/mL) was added into RAW264.7 cells for screening with changing culture medium every 2-3 days. At last, the cells in the control non-infected group were all dead and the drug screening was completed. The screened RAW264.7 cells were named Tet-PCNA KD cells.

### Cell counts

The effects of PCNA knockdown on cell proliferation were determined by cell counts. Tet-PCNA KD cells were seeded into 96-well plate and treated with Tet (20 μg/mL) or RANKL (100 ng/mL) as indicated. After 1, 2 or 3 days of incubation, the cells were trypsinized and counted with Trypan blue staining on an inverted microscope.

### Immunofluorescence assay

The immunofluorescence (IF) assay was performed on RAW264.7 cells as described previously [12, 48]. Briefly, the primary antibodies against PCNA (1:50 dilution, Proteintech, USA), Rab7 (1:50 dilution, Proteintech, USA), LAMP1 (1:50 dilution, Proteintech, USA), LC 3 (1:100 dilution, Cell Signaling Technology, USA) and secondary antibodies conjugated with Alexa Fluor 488 and Alexa Fluor 594 dyes (Cat. no. ZF-0513, ZF-0511, and ZF-0512; ZSGB-BIO, China) were used for the test. To observe the superstructures of F-actin, phalloidine conjugated with Alexa Fluor 594 (Sigma, USA) was used to stain the cells for 1 hour at room temperature. The morphology of cell nuclei was shown by DAPI (Sigma, USA) staining for 10 min. The imaging experiments were digitized on laser scanning confocal microscopes (LSM700, Zeiss, Jena, Germany) as described previously [12, 48]. In addition, the z-stacks plug-in was used to perform the consecutive scans of the cells in the direction of Z-axis.

### Tartrate-resistant acid phosphatase (TRAP) staining

TRAP staining was performed as described previously [12]. Briefly, RAW264.7 cells cultured in 96-well plate was fixed with 4% Paraformaldehyde at room temperature for 20 min. After washing, the cells were stained with TRAP staining solution at 37 °C for about 20 min with close observation. Then, the TRAP staining solution was removed and the cells were washed twice with PBS. Lastly, the PBS containing sodium azide was used to preserve the samples. Samples were observed by an Olympus Fluoview 500 microscope (Japan).

### Quantitative RT-PCR assay

After transfection with siRNAs of PCNA or the control NC coupled with or without RANKL (100 ng/mL, 3 days) induction, total RNA of RAW264.7 cells was extracted with trizol (Invitrogen). Then the reverse transcription (RT) was performed as described previously [12] with iScript™ cDNA Synthesis Kit (Bio-Rad). RT-PCR was carried out using SsoFast EvaGreen Supermix (Bio-Rad) on a qRT-PCR system (Mini Opticon, Bio-Rad). The following specific primers were used: PCNA, 5’-CCTGCTGGGATATTAGCT-3’ (forward) and 5’-AACTTTCTCCTGGTTTGG-3’ (reverse); NFATc1, 5’-TGCTCCTCCTCCTGCTGCTC-3’ (forward) and 5’-CGTCTTCACCTCCACGTCG-3’ (reverse); GAPDH, 5’-TCACCATCTTCCAGGAGCG-3’ (forward) and 5’-CTGCTTACCACCTTCTTGA-3’ (reverse). GAPDH was used as the internal control.

### Nuclear, cytoplasmic or membrane protein extraction

RAW264.7 cells were treated with RANKL (100 ng/mL) for three days, then subjected to the extraction of nuclear, cytoplasmic and/or membranal proteins. Extraction buffer includes 10 mmol/L Tris-HCl, 10 mmol/L KCl, 5 mmol/L MgCl2 (PH 7.6), 1% protease inhibitor cocktail (Roche) and protease inhibitors including 1 mM PMSF, 10 mM NaF, 1 mM Na3VO4. Nuclear isolation buffer includes 10 mmol/L Tris-HCl, 10 mmol/L KCl, 5 mmol/L MgCl2, and 0.35 mol/L Sucrose. Firstly, 250 μL extraction buffer was added to the cells. Then, the cell membrane was ruptured with 0.6% Triton X-100 incubation for 30 min on ice. 250 μL nuclear isolation buffer was added to carry out density gradient centrifugation for 10 min with 600 g. After that, the cytoplasmic components were in the supernatant, and the nuclear fractions were in the precipitation. The precipitation was dissolved in 50 μL SDS lysis buffer (Beyotime, China) containing 1% protease inhibitor cocktail (Roche) and acted las nuclear fractions. Next, the supernatant was centrifuged for another 30 min at 4 ^o^C with 10000 g to differentiate cytoplasmic and membrane proteins. The produced supernatant of cytoplasmic proteins was transferred into another centrifuge tube, added with four times of volume pre-cold acetone and incubated at -20 ^o^C overnight. Then, the supernatant was centrifuged again with 10000 g at 4 ^o^C for 30 min and produced precipitation dissolved in 200 μL SDS lysis buffer (Beyotime, China) containing 1% protease inhibitor cocktail (Roche) was the final cytoplasmic fractions. The concentrations of the isolated proteins were examined by BCA assay.

### Co-immunoprecipitation (co-IP) assay

The cytoplasmic fractions of RAW264.7 cells with or without RANKL induction were obtained using above method of nuclear and cytoplasmic protein extraction. Then, the cytoplasmic fractions were performed with PCNA antibody or the control IgG according to the standard protocols described previously [12, 49]. About 2 μg PCNA antibody was mixed with 1 mg total protein from the cytoplasmic fractions of RAW264.7 cells. The immune complexes were performed for subsequent in-solution digestion and LC-MS/MS analysis, or separated by western blotting using PCNA and β-actin antibodies.

In the subsequent experiments of verifying the interaction between PCNA and β-actin, RAW264.7 cells with or without RANKL (100 ng/mL) treatment for 3 days were used to perform co-IP assay. The control IgG and primary PCNA antibodies were used and the immune complexes were separated by SDS-PAGE followed by western blotting.

### Western blotting assay

The cells after the corresponding treatments were harvested and lysed with lysis buffer (20 mM Tris (pH7.5), 150 mM NaCl, 1 % Triton X-100, sodium pyrophosphate, β-glycerophosphate, EDTA, Na3VO4, leupeptin, and 1 % protease inhibitor cocktail (Roche)). 10 % or 12 % SDS-PAGE was used to separate the collected cell lysates according to the methods described previously [12, 48].

Blots were probed with the specific antibodies against PCNA (Proteintech, Chicago, USA), β-actin (Proteintech, Chicago, USA), Lamin A/C (Proteintech, Chicago, USA), Tubulin (Proteintech, Chicago, USA), atp A1 (Cell Signaling Technology, USA). Secondary antibodies conjugated with Horseradish peroxidase (ProteinTech Group) and enhanced chemiluminescence (ECL kit, Beyotime, China) were employed to test the expression of proteins.

### Tryptic in-gel digestion and LC-MS/MS analysis

After silver staining of SDS-PAGE gels of separating the immune complexes bound to PCNA antibody or the control IgG, the gel slices in the whole PCNA lane or whole IgG lane were extracted and collected into several tubes. The gel slices with the same electrophoretic mobility were marked with the same numbers. Then, tryptic in-gel digestion of these gel slices to generate peptide mixtures were performed as described previously [50].

LC-MS/MS analysis was performed on the produced peptide mixtures on the Orbitrap Fusion Lumos (Thermo) mass spectrometer operated in a data-dependent mode as described previously [51].

Raw data were automatically processed by MaxQuant 1.1.1.2 software against a database of Uniprot-Mus (20181009) with the default settings, including precursor-ion mass tolerance of 30 ppm and a fragment-ion mass tolerance of 0.6 Da. The false discovery rate for proteins and peptides determination was set to 0.01. The Max Missed Cleavages was set to 2.

### Cytoscape software platform analysis

Gene Ontology (GO) enrichment analysis and KEGG Pathways analysis of the produced differential proteins of PCNA lane compared with the control IgG lane were performed with ClueGO plug-in in Cytoscape v 3.6.1 software (https://cytoscape.org/). ClueGO is a Cytoscape plug-in that annotates proteins with GO terms [52]. The corresponding p-values of GO terms and KEGG were analyzed by the ClueGO plug-in of Cytoscape.

### *In vitro* bone resorption assay

Tet-PCNA KD cells (RAW264.7 cells with a Tet-on inducible PCNA knockdown) were seeded at 5000 cells/well on the Corning Osteo Assay Surface multiple well plates (Corning® Osteo Assay Surface, Corning, USA). 12 h later, the cells were simultaneously treated with Tet (20 μg/mL), RANKL (100 ng/mL) and M-CSF (50 ng/mL) for 10 days, changing the medium every three days, to induce the knockdown of PCNA, differentiation of osteoclasts and osteoclast-mediated bone resorption on the synthetic surface of Corning multiple well plates. Tet-PCNA KD cells treated only with RANKL and M-CSF but not with Tet were set as control. Then, 100 μL of 10% bleach solution was added to the surface of each well for 5 min at room temperature according to the manufacturer’s instructions. Individual resorption pits or multiple pit clusters were observed using an Olympus Fluoview 500 microscope (Japan) at 25x magnification. ImageJ software was used to analyze the area of total pits.

### GST pull-down assay

His-PCNA plasmid was constructed previously in our lab [49]. GST-β-actin was constructed through amplification of β-actin cDNA from RAW264.7 cells and ligation in-frame into the pGEX vector. The expressions of two recombinant proteins of His-PCNA and GST-β-actin *in vitro* were obtained as described previously [49]. Then *in vitro* GST pull-down assay with an equal amount of GST or GST-β-actin (20 μg) mixed with 20 μg His-PCNA protein was performed in lysis buffer A for 4 h as described previously [49]. The pellets of GST or GST-β-actin beads were subjected to western blot analysis. The PCNA antibody and the CBB staining of SDS-PAGE gel were applied to analyze the presence of His-PCNA in the pellets and the amounts of recombinant proteins, respectively.

### Statistics analysis

Statistical analyses were carried out by Student’s t-test and the statistical significance was defined as p<0.05.

## Supplementary Material

Supplementary Figure 1

Supplementary Table 1
