# Supplementary material for: Cytoplasmic PCNA is located in the actin belt and involved in osteoclast differentiation
Source: Aging (Albany NY). 2020 Jun 27;12(13):13297–317. doi: 10.18632/aging.103434 (PMC7377826; doi:10.18632/aging.103434)
Supplement: Supplementary Figure 1 [file aging-12-103434-s002..pdf]

SUPPLEMENTARY FIGURE

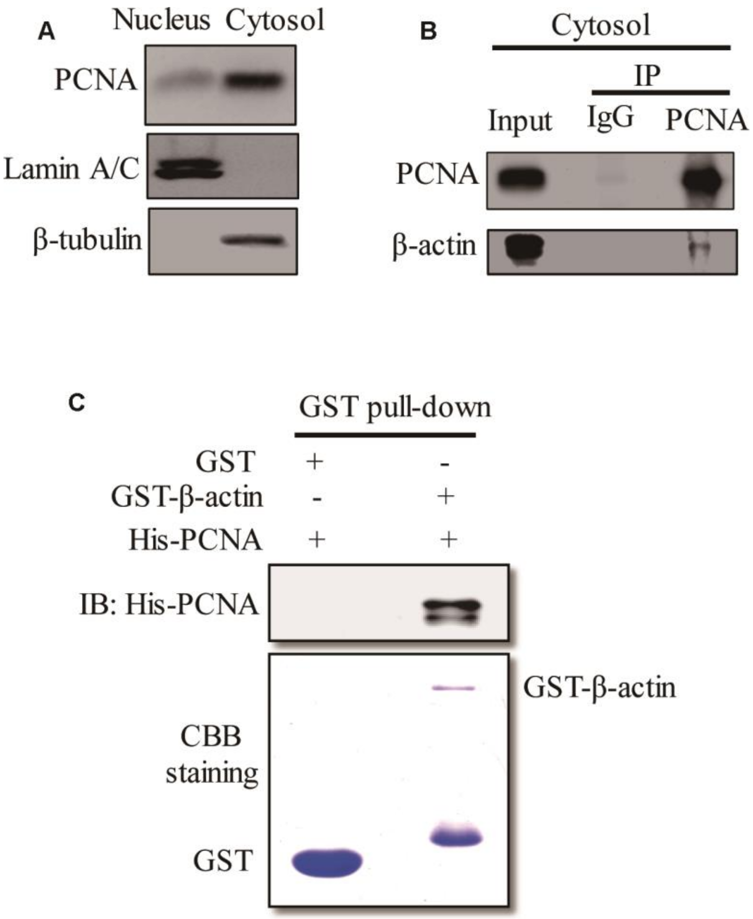

Supplementary Figure 1. The confirmation of β-actin interacting with cytoplasmic PCNA. (**A**) RAW264.7 cells after RANKL (100 ng/mL) induction for three days were carried out fractionation experiment to separate the nuclear and cytoplasmic fractions. The expression of PCNA in both fractions was examined by western blotting. Lamin A/C and β-tubulin were employed as markers for nuclear and cytoplasmic fractions, respectively. (**B**) The cytoplasmic fraction of A was applied to perform co-IP assay using IgG and primary PCNA antibody, respectively. Western blot assay was then carried out to detect PCNA and β-actin in the immunocomplex. (**C**) PCNA bound to β-actin directly in a cell-free GST pull-down assay. The proteins bound to the pellets of GST or GST-β-actin were analyzed by IB with PCNA antibody.
