# Supplementary material for: Cytoplasmic PCNA is located in the actin belt and involved in osteoclast differentiation
Source: Aging (Albany NY). 2020 Jun 27;12(13):13297–317. doi: 10.18632/aging.103434 (PMC7377826; doi:10.18632/aging.103434)
Supplement: Supplementary Table 1 [file aging-12-103434-s001..docx]

**Supplementary Table 1. Total 76 proteins interacting with cytoplasmic PCNA identified by LC-MS/MS.**

| Protein IDs | Protein Descriptions | Gene names | Peptides | Sequence coverage [%] | Mol. Weight [kDa] | Score |
| --- | --- | --- | --- | --- | --- | --- |
| P60710 | Actin, cytoplasmic 1 | ACTB | 11 | 44.3 | 41.736 | 323.31 |
| P63260 | Actin, cytoplasmic 2 | ACTG1 | 11 | 44.3 | 41.736 | 323.31 |
| P20029 | 78 kDa glucose-regulated protein | HSPA5 | 7 | 15 | 72.421 | 102.78 |
| Q02257 | Junction plakoglobin | JUP | 7 | 12.9 | 81.8 | 54.104 |
| P68372 | Tubulin beta-4B chain | TUBB4B | 6 | 18 | 49.83 | 52.237 |
| Q9D6F9 | Tubulin beta-4A chain | TUBB4A | 6 | 18 | 49.83 | 52.237 |
| E9Q557 | Desmoplakin | DSP | 6 | 2.6 | 332.91 | 40.225 |
| S4R257 | Glyceraldehyde-3-phosphate dehydrogenase | GAPDH | 5 | 28.9 | 29.939 | 33.447 |
| Q6PGB6 | N-alpha-acetyltransferase 50 | NAA50 | 1 | 8.3 | 19.414 | 24.36 |
| Q9QYX7 | Protein piccolo | PCLO | 1 | 0.2 | 550.83 | 18.709 |
| A0A0U1RNQ6 | Elongation factor Tu, mitochondrial | TUFM | 1 | 11.9 | 12.929 | 17.691 |
| P99024 | Tubulin beta-5 chain | TUBB5 | 5 | 15.3 | 49.67 | 16.393 |
| Q9CWF2 | Tubulin beta-2B chain | TUBB2B | 2 | 6.1 | 49.67 | 15.639 |
| Q7TMM9 | Tubulin beta-2A chain | TUBB2A | 2 | 6.1 | 49.67 | 15.639 |
| P97868 | E3 ubiquitin-protein ligase RBBP6 | RBBP6 | 1 | 0.7 | 199.59 | 15.418 |
| Q8C605 | ATP-dependent 6-phosphofructokinase | PFKP | 2 | 3.8 | 85.546 | 14.995 |
| P11152 | Lipoprotein lipase | LPL | 1 | 4 | 53.109 | 14.993 |
| E9Q3D6 | Heat shock protein HSP 90-beta | HSP90B1 | 2 | 15.3 | 22.482 | 13.867 |
| Q2EMV9 | Poly [ADP-ribose] polymerase 14 | PARP14 | 2 | 2.3 | 203.8 | 11.317 |
| P19157 | Glutathione S-transferase P | GSTP | 2 | 15.2 | 23.609 | 11.291 |
| Q9WUM5 | Succinate--CoA ligase [ADP/GDP-forming] subunit alpha, mitochondrial | SUCLGL | 1 | 3.5 | 36.154 | 11.09 |
| Q922F4 | Tubulin beta-6 chain | TUBB6 | 3 | 9.2 | 50.09 | 10.503 |
| Q3UGF1 | WD repeat-containing protein 19 | WDR19 | 1 | 0.9 | 151.46 | 9.8982 |
| A0A1B0GSU4 | Growth hormone-regulated TBC protein 1 | GRTP1 | 2 | 6.6 | 22.526 | 9.502 |
| Q3UV17 | Keratin, type II cytoskeletal 2 oral | KRT76 | 2 | 2.7 | 62.844 | 9.2963 |
| Q5SV02 | DNA repair protein RAD50 | RAD50 | 1 | 1.6 | 153.54 | 9.2719 |
| P14869 | 60S acidic ribosomal protein P0 | RPLP0 | 1 | 9.8 | 34.216 | 8.1519 |
| F8VQ79 | Myosin-IIIb | MYO3B | 1 | 1.3 | 151.1 | 7.699 |
| Q78ZA7 | Nucleosome assembly protein 1-like 4 | NAP1L4 | 1 | 2.9 | 42.679 | 7.5992 |
| A0A0M3HEP7 | Phospholipid-transporting ATPase | ATP8A1 | 1 | 0.7 | 131.51 | 7.5863 |
| Q8BFT9 | Synaptic vesicle 2-related protein | SVOP | 1 | 1.5 | 60.768 | 7.5686 |
| P0C6F1 | Dynein heavy chain 2, axonemal | DNAH2 | 1 | 0.4 | 511.56 | 7.5637 |
| Q91V41 | Ras-related protein Rab-14 | RAB14 | 1 | 4.2 | 23.897 | 7.3836 |
| A0A0N4SW38 | Olfactory receptor 1395 | OLFR1395 | 1 | 3.3 | 27.229 | 7.204 |
| Q91W43 | Glycine dehydrogenase (decarboxylating), mitochondrial | GLDC | 1 | 1.9 | 113.27 | 7.1861 |
| D3YWG7 | DCN1-like protein | DCUN1D3 | 1 | 4.8 | 18.213 | 7.0288 |
| A0A0A6YWY1 | TBC1 domain family member 8 | TBC1D8 | 1 | 1 | 115.86 | 6.8453 |
| B1AXW4 | Peroxiredoxin-1 | PRDX1 | 1 | 8.1 | 13.632 | 6.7665 |
| Q80UW5 | Serine/threonine-protein kinase MRCK gamma | CDC42BPG | 1 | 0.7 | 172.14 | 6.6938 |
| P62717 | 60S ribosomal protein L18a | RPL18A | 1 | 7.4 | 20.732 | 6.6842 |
| E9PZ92 | Exocyst complex component 5 | EXOC5 | 1 | 1.1 | 74.225 | 6.6569 |
| Q6ZWV7 | 60S ribosomal protein L35 | RPL35 | 1 | 8.1 | 14.552 | 6.5383 |
| A0A1B0GQW7 | Folate receptor beta | FOLR2 | 1 | 24.1 | 9.9813 | 6.4427 |
| Q7TSY8 | Shugoshin 2 | SGO2 | 1 | 1.4 | 130.28 | 6.4281 |
| B2RXA7 | Cytochrome P450, family 26, subfamily c, polypeptide 1 | CYP26C1 | 1 | 5.6 | 57.025 | 6.4182 |
| G5E832 | Adenomatosis polyposis coli 2 | APC2 | 1 | 0.6 | 243.11 | 6.3311 |
| Q5U458 | DnaJ homolog subfamily C member 11 | DNAJC11 | 1 | 1.3 | 63.232 | 6.2507 |
| D3Z6F5 | ATP synthase subunit alpha | ATP5A1 | 1 | 2.6 | 54.594 | 6.2155 |
| Q8K1K2 | 26S protease regulatory subunit 8 | PSMC5 | 1 | 3.7 | 38.751 | 6.2142 |
| Q3USB7 | Inactive phospholipase C-like protein 1 | PLCL1 | 1 | 1 | 122.67 | 6.2113 |
| P62855 | 40S ribosomal protein S26 | RPS26 | 1 | 13 | 13.015 | 6.1895 |
| Q8CDD9 | Ligand-dependent nuclear receptor-interacting factor 1 | LRIF1 | 1 | 1.9 | 82.997 | 6.1306 |
| P63017 | Heat shock cognate 71 kDa protein | HSPA8 | 3 | 6.7 | 68.778 | 6.0869 |
| Q61171 | Peroxiredoxin-2 | PRDX2 | 1 | 5.6 | 21.778 | 6.0768 |
| Q08331 | Calretinin | CALB2 | 1 | 4.1 | 31.372 | 6.0233 |
| A0A0J9YUC3 | Cytosolic carboxypeptidase-like protein 5 | AGBL5 | 1 | 6.2 | 20.136 | 6.0038 |
| P62806 | Histone H4 | HISTLH4A | 1 | 9.7 | 11.367 | 5.9863 |
| Q6RHW0 | Keratin, type I cytoskeletal 9 | KRT9 | 1 | 3.1 | 72.494 | 5.9828 |
| D3YWU8 | tRNA pseudouridine synthase A, mitochondrial | PUS1 | 1 | 11.6 | 16.346 | 5.9804 |
| A0A087WNU5 | Ankyrin-3 | ANK3 | 1 | 0.5 | 315.42 | 5.9773 |
| F8VPT6 | Ecotropic viral integration site 5 protein | EVI5 | 1 | 1.4 | 92.9 | 5.9661 |
| Q61909 | Protein CBFA2T1 | RUNXLT1 | 1 | 5.7 | 64.337 | 5.9639 |
| A2AGT5 | Cytoskeleton-associated protein 5 | CKAP5 | 1 | 0.7 | 218.71 | 5.9414 |
| A0A0G2JEK1 | Phosphatidylinositol 4-kinase type 2-beta | PI4K2B | 1 | 19.1 | 9.8176 | 5.8618 |
| Q8CGA0 | Protein phosphatase 1F | PPM1F | 1 | 3.3 | 49.61 | 5.8157 |
| P56480 | ATP synthase subunit beta, mitochondrial | ATP5B | 1 | 4 | 56.3 | 5.81 |
| D3YV69 | Ras-related protein Rab-6A | RAB6A | 1 | 6.3 | 19.961 | 5.8047 |
| P61294 | Ras-related protein Rab-6B | RAB6B | 1 | 6.3 | 19.961 | 5.8047 |
| Q8BHD0 | Ras-related protein Rab-39A | RAB39A | 1 | 6.3 | 19.961 | 5.8047 |
| Q9CZN7 | Serine hydroxymethyltransferase | SHMT2 | 1 | 2.2 | 55.758 | 5.8018 |
| E9QAT6 | CAD protein | CAD | 1 | 1.3 | 105.68 | 5.7965 |
| Q8BTU6 | Eukaryotic initiation factor 4A-II | EIF4A2 | 1 | 2.2 | 41.29 | 5.7921 |
| A0A0R4J0E4 | Integrator complex subunit 7 | INTS7 | 1 | 1.4 | 106.87 | 5.7772 |
| H3BK80 | PH and SEC7 domain-containing protein 2 | PSD2 | 1 | 1.8 | 59.919 | 5.7702 |
| A2A547 | Ribosomal protein L19 | RPL19 | 1 | 8.8 | 23.247 | 5.7663 |
| Q5GH67 | XK-related protein 4 | XKR4 | 1 | 7 | 71.502 | 5.7563 |
